# Real-world experience of intravitreal faricimab injection in previously treated neovascular age-related macular degeneration eyes: a case series

**DOI:** 10.1186/s12886-025-03953-9

**Published:** 2025-03-10

**Authors:** Maria A. Bantounou, Mohammed Elsheikh, Adelehin Ijasan, Cynthia Santiago

**Affiliations:** 1 School of Medicine, University of Aberdeen, Aberdeen, UK; 2 Department of Ophthalmology, Aberdeen Royal Infirmary, Aberdeen, AB25 2ZN UK

**Keywords:** Anti-vascular endothelial growth factor, Faricimab, Vabysmo, Intravitreal injection, Neovascular age-related macular degeneration, Switch therapy

## Abstract

**Background:**

Faricimab is a novel anti-vascular endothelial growth factor agent, used to treat patients with neovascular age-related macular degeneration (nAMD). This study assessed efficacy and safety of faricimab in previously treated eyes.

**Methods:**

This retrospective study included previously treated nAMD patients who had received at least three faricimab injections. Baseline data were collected from February 2023 to September 2023, and follow-up data were collected until April 2024. The patients were divided into two cohorts: (1) the "Loaded" cohort, which received four weekly injections prior to treatment extension, and (2) the "Interval-Matched" cohort, which continued on the same treatment interval as their previous regimen. Efficacy was evaluated based on the primary outcome measures: central subfield thickness (CST), the presence of macular fluid, and visual outcomes. Safety was assessed through the secondary outcome measure of adverse event reporting.

**Result:**

Two hundred thirty-seven participants (297 eyes) were included with a mean age of 80.7 ± 7 years, 44% were males. 2,237 faricimab injections were administered (7.5 ± 1.9 per eye). In the loaded cohort, CST decreased from 315.1 ± 86.0 µm to 288.0 ± 63.6 µm (*p* < 0.001). The percentage of dry macula increased from 11.0% to 42.5% (*p* < 0.001). Vision changed from 67.9 ± 12.3 to 69.3 ± 13.4 letters (*p* = 0.002), and the injection interval extended from 5.3 ± 1.3 to 6.4 ± 2.1 weeks (*p* < 0.001). For the interval-matched cohort, CST decreased from 302.8 ± 57.4 µm to 291.2 ± 62.6 µm (*p* = 0.001). The percentage of dry macula increased from 22.5% to 47.7% (*p* < 0.001). Vision changed from 65.9 ± 13.8 to 65.0 ± 17.1 letters (*p* = 0.613), and the injection interval extended from 6.6 ± 2.8 to 7.9 ± 3.2 weeks (*p* < 0.001). 68 (28.7%) adverse events were reported, of which 9 (3.8%) were serious.

**Conclusion:**

Faricimab showed beneficial anatomical response with stable vision, and less injections. The loaded cohort exhibited superior outcomes but needed more injections.

**Supplementary Information:**

The online version contains supplementary material available at 10.1186/s12886-025-03953-9.

## Background

Neovascular age-related macular degeneration (nAMD) may lead to irreversible vision loss if not identified and treated early [1, 2]. The advent of intravitreal anti-vascular endothelial growth factor (anti-VEGF) agents has marked a significant paradigm shift in managing patients with nAMD [3]. At present, typical practices include intravitreal injections at treatment intervals ranging from four to sixteen weeks [4]. In clinical practice only a small percentage of patients achieve treatment intervals of 12 weeks or more [5]. Frequent intravitreal injections undoubtedly result in a significant treatment burden for both patients and clinicians [4]. This burden is continuously recognised as a significant unfulfilled need in the management of nAMD.

Additionally, various hindrances in managing nAMD in real-world settings have been identified. Obstacles such as repeated and frequent injections, adverse effects sustained during intravitreal anti-VEGF therapy, negative patient attitude towards this treatment, medical comorbidities and difficulties securing regular patient transport to clinics can all result in delayed intravitreal injections and thus significantly affect outcomes [6].

New anti-VEGF agents are constantly being developed and introduced into clinical practice to tackle this treatment burden. They aim to increase treatment durability, while maintaining safety and achieving good anatomical and functional outcomes, thereby improving the overall management of nAMD [7]. Faricimab is the latest anti-VEGF agent to be approved for use in nAMD [8]. It is a novel, bispecific, monoclonal antibody that targets both VEGF-A and angiopoietin-2, crucial in the pathogenesis of AMD. The simultaneous suppression of these factors by faricimab may result in a more complete and enduring prevention of neovascularisation and exudation-forming pathways. This is proposed to produce superior anatomical and functional outcomes compared to previous anti-VEGF treatments [9]. This has been reflected by the results of the TENAYA and LUCERNE trials, which are randomised, double-masked, non-inferiority trials which evaluated the efficacy, durability, and safety of intravitreal faricimab for nAMD [10]. Results from these trials produced non-inferior visual outcomes with faricimab dosing up to Q16W compared to aflibercept dosing Q8W. This demonstrated faricimab’s potential to meaningfully extend time intervals between treatments whilst sustaining its efficacy [10].

In this study, we aimed to measure real world outcomes by assessing the efficacy and safety of intravitreal faricimab therapy in previously treated nAMD eyes of patients attending a large tertiary hospital. We evaluated anatomical and functional measures and the total number of adverse events reported by patients.

## Methods

### Study design and setting

This was a retrospective single-centre case series, undertaken at the Department of Ophthalmology, Aberdeen Royal Infirmary, Aberdeen, United Kingdom, a tertiary centre covering a population of approximately 600,000 and seeing an average of 250 new nAMD patients per year.

### Participant characteristics

We included participants with active nAMD that: 1) were treated with another anti-VEGF prior to switch to faricimab, 2) had an inadequate response to previous anti-VEGF, defined as persistent disease activity [presence of any amount of intraretinal and/ or subretinal fluid on optical coherence tomography (OCT) three months or more after the loading phase] or required frequent injections (four weekly injections three months or more after the loading phase) to maintain a dry macula and 3) received three or more intravitreal faricimab injections. Baseline data were collected from February 2023 to September 2023, and follow-up data were collected until April 2024.

We excluded patients: 1) without baseline data (e.g. due to transfer from another health board), 2) without follow-up OCT or OCT not possible (e.g. hazy due to cataracts) or practical (e.g. patients unable to undertake scan due to physical or mental health limitations) and 3) that received a different anti-VEGF after switch to faricimab.

### Faricimab treatment protocol

In our centre, patients were commenced on faricimab in two ways:

1. Loaded cohort: Participants who received faricimab as per the TENAYA and LUCRENE trials protocol [10] (minimum of 4 doses at 4-week intervals before potentially extending treatment); including those that experienced delays of a maximum total of 2 weeks in administration during the loading period, due to real-world factors such as missed appointments, delays, or patient preferences.
2. Interval-matched: Participants who received faricimab either to align with their previous anti-VEGF administration interval or at intervals longer than the standard 4-week loading schedule for the first 4 doses, even with the additional 2-week grace period.

All patients underwent at baseline a comprehensive eye examination; assessment of visual acuity using the Early Treatment of Diabetic Retinopathy Study (ETDRS) letter score, intraocular pressure measurement, anterior segment assessment and dilated fundoscopy. Patients underwent Optos colour imaging, spectral-domain OCT (Spectralis; Heidelberg Engineering, Heidelberg, Germany) with cube scanning and OCT angiography at first presentation; OCT scans were repeated at subsequent visits. Repeat fundus photos, OCT angiography, fundus fluorescein angiogram and indocyanine green angiography were performed as per physician discretion to aid with diagnosis or treatment.

Intravitreal faricimab injections were administered by trained allied healthcare professional and ophthalmologists, as per the Royal College of Ophthalmologists guidance [11].

### Data collection

The anonymised data were retrieved from the electronic medical records (EMR) system Medisoft Ophthalmology (Medisoft Limited, Leeds, UK). Prospective guidelines were established for inputting data into the EMR for nAMD patients, enhancing the reliability and reproducibility of the dataset.

The collected data included: age, sex, ethnicity, best corrected visual acuity (VA), Central subfield thickness (CST), presence of macular fluid [dry, intraretinal fluid (IRF), subretinal fluid (SRF), both], prior anti-VEGF treatment, number of total injections prior to switch, number of administered injections, injection interval prior to switch, follow-up time, adverse events.

Data from the OCT images were independently collected by two authors (MB and ME) and then compared. In cases of discrepancy, a third author (AI) made the final determination.

### Aims and outcome measures

The primary aim of our study was the efficacy of faricimab. Anatomical response was evaluated by the CST, presence of fluid (SRF, IRF, both, dry) and changes in the presence of fluid from baseline to the final follow-up OCT scan (improved, stable, worse). The OCT was classified as dry when neither SRF nor IRF was detected. Functional response was evaluated by the patient’s VA using the ETDRS letter score. These parameters were collected at baseline (at the time of switch to faricimab), at the most recent follow-up clinical encounter and at the 3-, 6-, 9- and 12-month follow-up intervals. The secondary aim, safety, was assessed by adverse events reporting.

### Statistical analysis

Statistical analysis was conducted using RStudio 2023 with R version 4.3.1 [12]. For the analyses at prespecified timepoints, if data were unavailable precisely at the designated time point, the closest available data within a two-week window of the target date was utilized. Any remaining missing data were annotated as not available and then were omitted from the analysis. Continuous variables were reported as mean and standard deviation (SD). Categorical variables were described as counts and frequencies.

The paired T-test was employed for comparisons of paired normally distributed variables and the Wilcoxon signed ranks test if skewed. The McNemar test was performed to compare paired categorical variables. For independent comparisons with normally distributed variables the F-test was performed; for p-value > 0.05 an independent sample T test was undertaken, however, for p-value < 0.05 the Welch’s test was performed. If data were skewed, the Wilcoxon Man Whitney test was employed. For independent categorical variables the Chi-Square was used. A p-value of < 0.05 was considered statistically significant.

### Ethical considerations

This service evaluation was classed as an audit and thus did not require ethical permission (NHS Research Ethics Committee) [13]. The local quality improvement and assurance team registered the audit before data collection commenced. We conducted this study in accordance with the declaration of Helsinki, and the UK’s Data Protection Act.

## Results

### Participant demographics and baseline characteristics

Two hundred fifty-six patients and 339 eyes were started on faricimab between February 2023 and September 2023. Of these, 42 eyes were excluded from the analysis (Supplemental Digital Content, Fig. 1), resulting in a total of 237 participants with 297 treated eyes. Among these eyes, 153 (51.5%) were the right eye and 30 patients (*n* = 60 eyes, 20.0%) received bilateral treatment. 104 (44.0%) participants were male and 216 (91.1%) were of white ethnicity. The mean age was 80.7 ± 7.0.

Two hundred nine eyes were treated with one type of anti-VEGF therapy before the switch, 71 with two and 17 with three. Aflibercept was the most frequently prescribed anti-VEGF treatment (*n* = 275, 92.6%), followed by ranibizumab (*n* = 21, 7.1%) and broculizumab (*n* = 1, 0.3%). The mean number of intravitreal injections administered before the switch was 32.5 ± 24.4. Injections were administered on average every 6.0 ± 2.3 weeks.

At baseline, fluid on OCT was present in 83.2% of eyes, with 57.0% showing SRF, 12.0% indicating IRF, and 14.1% exhibiting both types of fluid on OCT. The baseline VA was 66.9 ± 13.1 letters.

Comparison of the baseline characteristics of the loaded and interval-matched cohorts (Table 1), revealed no statistically significant differences for relevant clinical characteristics. In the interval-matched cohort, 34 eyes (22.5%) were dry, compared to 16 eyes (11.0%) in the loaded cohort (*p*-value = 0.062). The mean CST at baseline was 302.8µm ± 57.4 in the interval-matched and 325.2 ± 97.2µm in the loaded cohort (*p*-value = 0.193). However, the interval-matched cohort had a longer injection interval of 6.6 ± 2.8 weeks compared to the loaded cohort's injection interval of 5.3 ± 1.3 weeks (*p*-value < 0.001).

**Table 1:** Baseline demographic and clinical characteristics of patients switched to faricimab, including comparison of baseline characteristics between loaded and interval-matched cohorts

	**Overall**	**Loaded**	**Interval-matched**	***P*-value**
Participants				
Number of patients	237	114	123	-
Participant age (years)	80.7 ± 7	80.4 ± 7	81 ± 7.1	0.491
Sex (male participants, %)	104 (43.9%)	51 (44.7%)	53 (43.1%)	0.901
Eyes				
Number of eyes	297	146	151	-
Laterality (right eye N, %)	153 (51.5%)	78 (53.4%)	75(49.7%)	0.645
Bilateral treatment	60 (20.2%)	-	-	-
Number of previous anti-VEGF therapies	1 prior IVT: 209 2 prior IVT: 71 3 prior IVT: 17	1 prior IVT: 97 2 prior IVT: 39 3 prior IVT: 10	1 prior IVT: 112 2 prior IVT: 32 3 prior IVT: 7	0.331
Number of previous injections	32.5 ± 24.4	33.9 ± 24	31.2 ± 24.7	0.3799
Injection interval prior to switch	6 ± 2.3	5.3 ± 1.3	6.6 ± 2.8	< 0.0001
Last anti-VEFG prior to switch: Aflibercept	275 (92.6%)	135 (92.5%)	140 (92.7%)	0.592
Last anti-VEFG prior to switch: Ranibizumab	21 (7.1%)	11 (7.5%)	10 (6.6%)	
Last anti-VEFG prior to switch: Brolucizumab	1 (0.34%)	-	1 (0.67%)	
Mean Visual Acuity	66.9 ± 13.1	67.9 ± 12.3	65.9 ± 13.8	0.301
Mean CST (µm)	313.8 ± 80.2	325.2 ± 97.2	302.8 ± 57.4	0.193
Fluid at baseline: Dry	50 (16.8%)	16 (11%)	34 (22.5%)	0.0623
Fluid at baseline: SRF	169 (56.9%)	90 (61.6%)	79 (52.3%)	0.733
Fluid at baseline: IRF	36 (12.1%)	16 (11%)	20 (13.2%)	1.000
Fluid at baseline: Both	42 (14.1%)	24 (16.4%)	18 (11.9%)	1.000

*Anti-VEGF* anti–vascular endothelial growth factor therapy, *IRF* intraretinal fluid, *N* number, *SRF* subretinal fluid

### Treatment summary

Two thousand two hundred thirty-seven faricimab injections (7.5 ± 1.9 per eye) were administered in our centre from February 2023 to April 2024. The mean injection interval between the final and penultimate faricimab injection was 7.2 ± 2.8 weeks. The switch allowed for an extension of the interval for 156 (52.5%) of the treated eyes. Over the average follow-up period of 9.6 ± 4.1 months, ten patients were switched back from faricimab to aflibercept; 4 due to lack of response, 4 due to adverse effects and 2 due to a drug administration error. A reduction in fluid was shown in 195 (70.6%) of the treated eyes.

The interval-matched cohort achieved a significantly longer injection interval of 7.9 ± 3.2 weeks compared to the loaded cohort (6.4 ± 2.1 weeks, *p*-value < 0.001). No significant differences were identified between the two cohorts in the overall OCT changes from baseline to the final OCT (*p*-value = 0.202) (Table 2).

**Table 2:** Descriptive outcomes of intravitreal faricimab for all patients and according to loading

	**Overall**	**Loaded**	**Interval-matched**	***P*-value**
Faricimab injections	7.5 ± 1.9	8.1 ± 1.9	7 ± 1.7	< 0.0001
Faricimab injection interval (weeks)	7.2 ± 2.8	6.4 ± 2.1	7.9 ± 3.2	< 0.0001
IVT interval extended (Y, %)	156 (52.5%)	72 (49.3%)	84 (55.6%)	0.0992
Switch from Faricimab to other IVT	10 (3.4%)	7 (4.8%)	3 (2%)	-
Follow up time (months)	9.6 ± 4.1	9.4 ± 2.3	9.9 ± 5.3	0.782
OCT overall change	Improved: 195 (70.6%) Stable: 57 (17.1%) Worse:45 (12.3%)	Improved: 103 (70.6%) Stable: 25 (17.1%%) Worse: 18 (12.3%)	Improved: 92 (60.9%) Stable: 32 (21.2%) Worse: 27 (17.9%)	0.202

*cohortIVT* intravitreal therapy, *OCT* optical coherence tomomgraphy, *Y* yes

### Anti-VEGF administration interval

Prior to switching to faricimab, 77 eyes were on 4-weekly injections, 141 eyes were on 5- and 6-weekly, 46 eyes on 7- and 8-weekly and 33 eyes were receiving injections at an interval > 8 weeks (Supplemental Digital Content, Table 1). After switching to faricimab, there was a reduction in the eyes receiving 4-weekly (*n* = 51 eyes, *p*-value = 0.021), and 5- and 6-weekly injections (*n* = 101, *p*-value = 0.010). A higher number of eyes received injections at an interval of > 8 weeks (*n* = 80 eyes, *p*-value < 0.001).

A higher proportion of eyes that were receiving previous anti-VEGF at longer injection intervals were subsequently interval-matched when switched to faricimab; 56.6% of eyes previously administered anti-VEGF at 7- and 8-week intervals and 87.9% at over 8-weekly intervals were interval-matched (Supplemental Digital Content, Table 1).

Among eyes previously receiving 4-weekly, 5- and 6-weekly and 7- and 8-weekly anti-VEGF therapy, an extension in the injection interval with faricimab was identified for 79.2%, 40.4%, and 23.9% of eyes, respectively. 51.5% of eyes previously treated at over 8-week intervals maintained their injection schedule (Supplemental Digital Content, Table 2).

### Structural and functional outcomes

Overall, the CST reduced from a mean of 325.2 ± 97.2µm at baseline to a mean of 291.5 ± 74.4µm (*p*-value < 0.001) at the last follow-up OCT available. The VA also improved, from a mean of 66.9 ± 13.1 at baseline, to a mean of 67.1 ± 15.5 (*p*-value = 0.016). The mean injection interval was extended to 7.2 ± 2.8 weeks from 6.0 ± 2.3 (*p*-value < 0.001). Following the transition to faricimab, a greater proportion of eyes were dry; 134 (45.1%) from 50 (16.8%) (*p*-value < 0.001). Evaluation of the same outcomes for the loading and interval-matched cohorts demonstrated a similar pattern of findings (Supplemental Digital Content, Table 3), with the exception of VA in the interval-matched cohort which decreased from 65.9 ± 13.8 at baseline to 65.0 ± 17.1 at the last faricimab injection (*p*-value = 0.613).

### CST changes over time

**Fig. 1:**
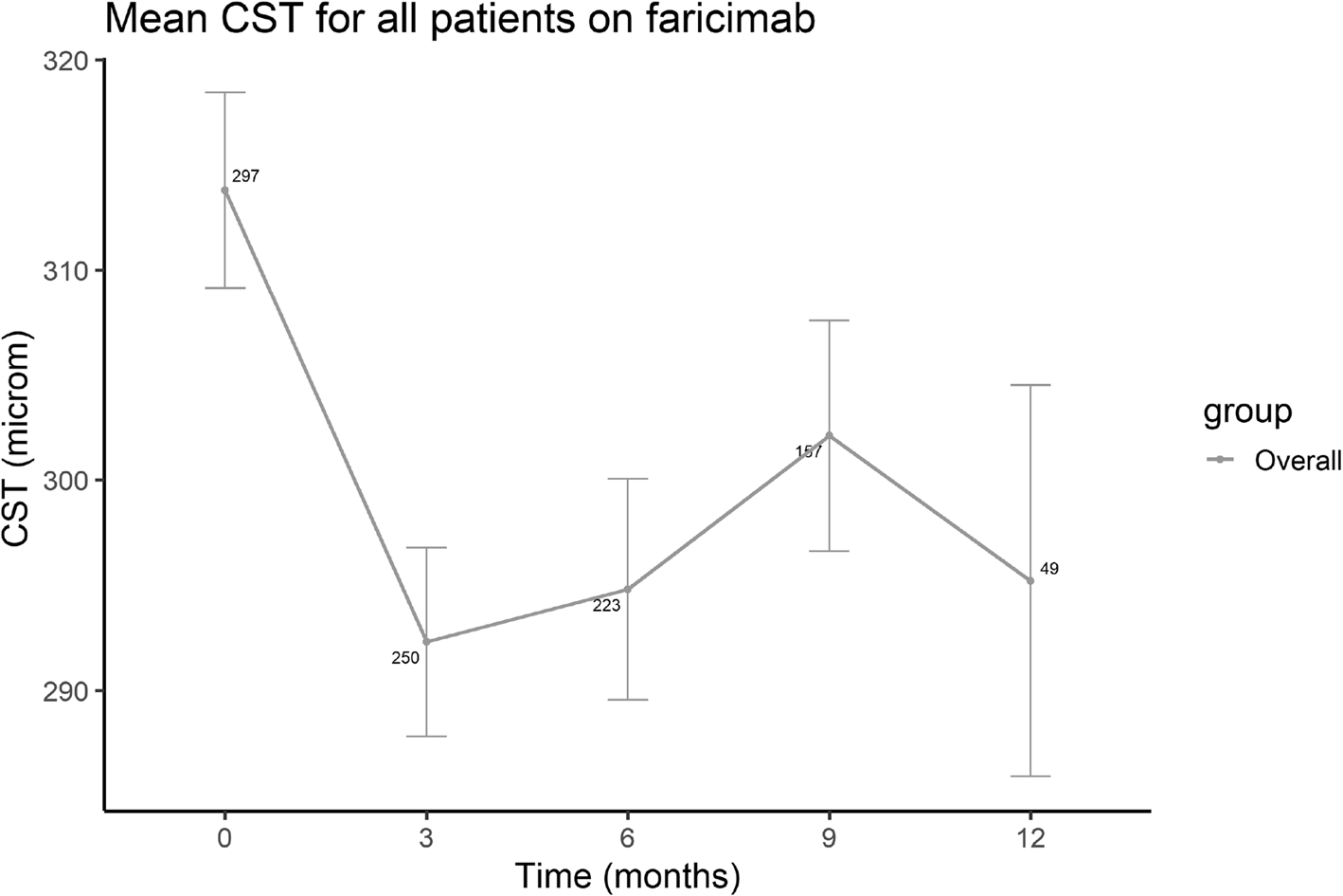
Line graph indicating the mean CST at baseline and after switching to faricimab at three, six, nine and 12 months for all treated patients. CST; central subfield thickness, microm; micrometer

**Fig. 2:**
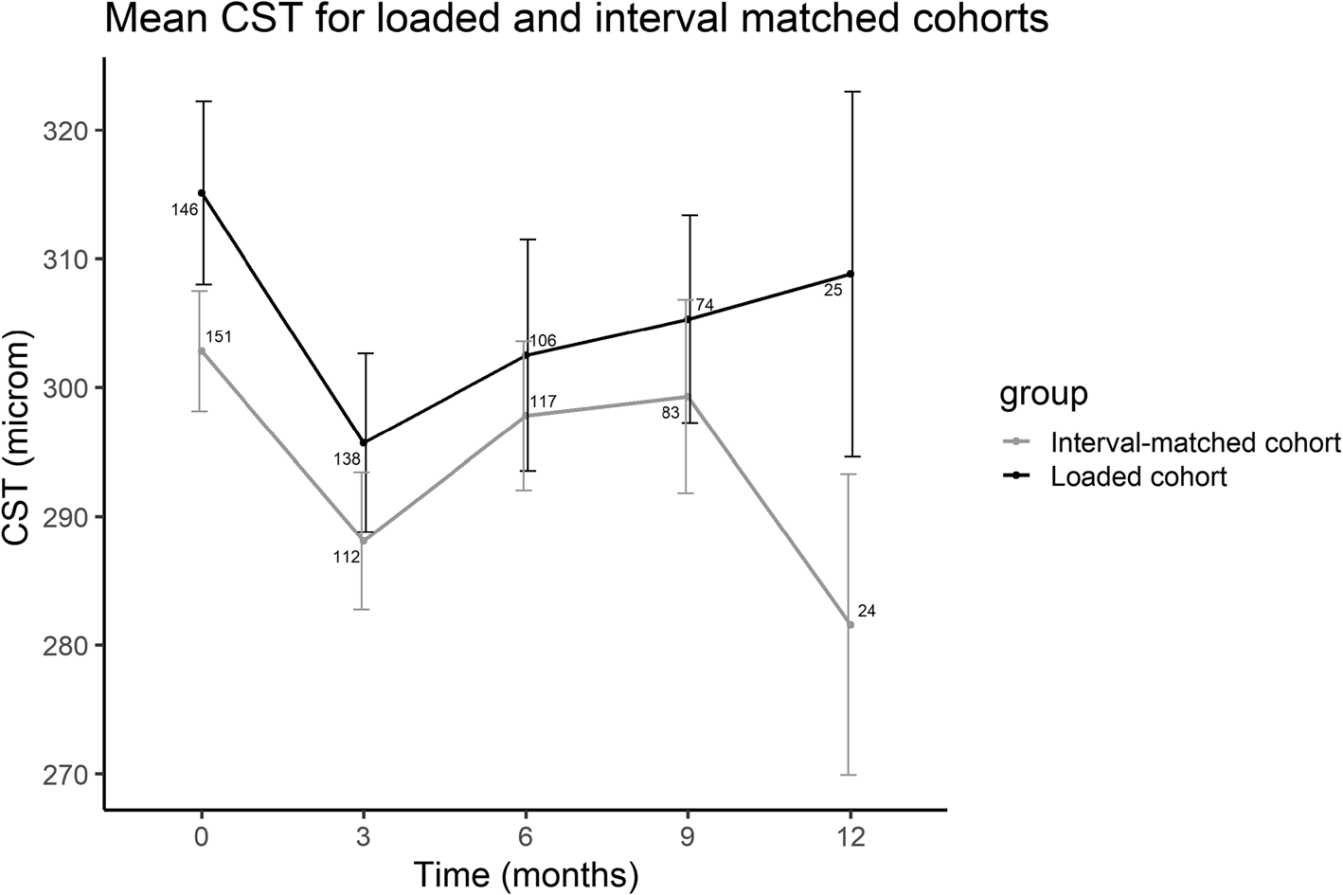
Line graph indicating the mean CST at baseline and after switching to faricimab at three, six and nine months for the loading phase and interval-matched cohorts. CST; central subfield thickness, microm; micrometer

Overall, the CST followed a downtrending pattern from 313.8 ± 80.2µm at baseline to 295.2 ± 65.2µm at 12 months (Fig. 1). The biggest CST decrease was at 3 months (Supplemental Digital Content, Table 4). Comparison of the loaded and interval-matched cohorts revealed similar trend lines during the initial 9 months of treatment, after which the CST trend lines of the two groups diverged at 12 months (Fig. 2); the CST of the loaded cohort increased, whereas the interval-matched cohort decreased.

### Visual acuity changes over time

**Fig. 3:**
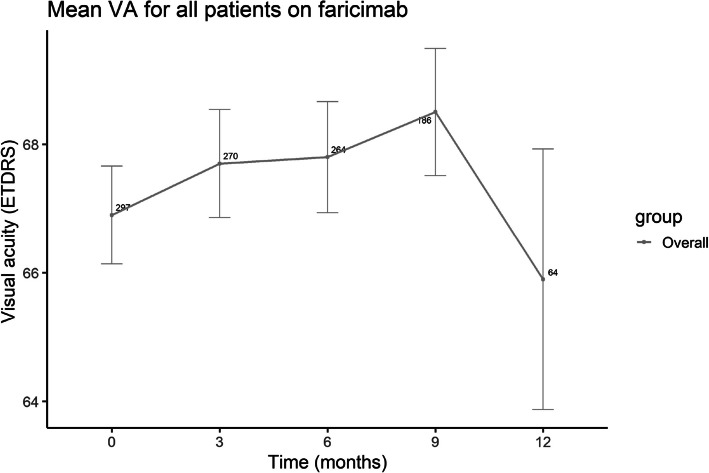
Line graph indicating the mean visual acuity (ETDRS) at baseline and after switching to faricimab at three, six, nine and 12 months for all participants treated with faricimab. ETDRS; Early Treatment Diabetic Retinopathy Study, VA; visual acuity

**Fig. 4:**
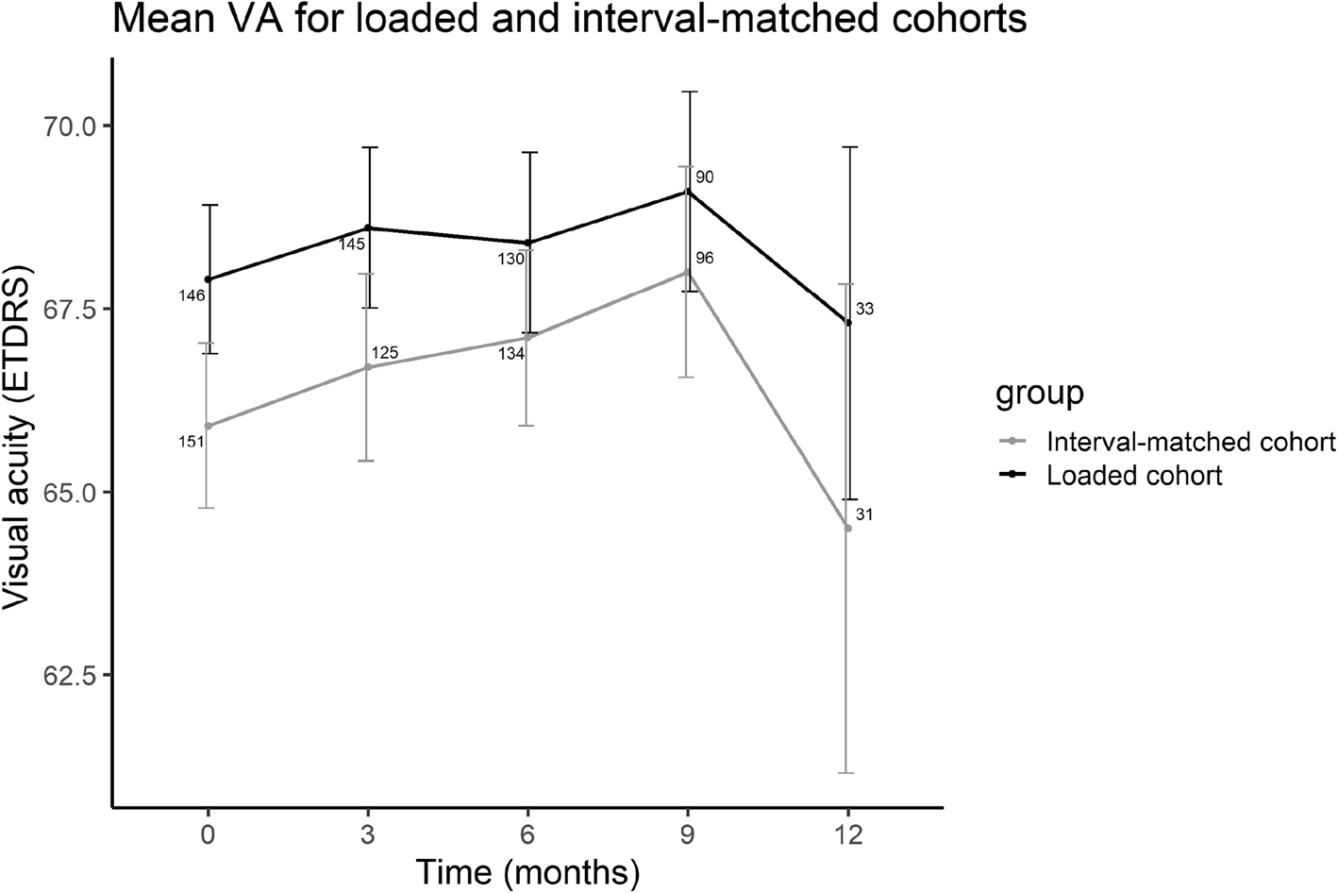
Line graph indicating the mean visual acuity (ETDRS) at baseline and after switching to faricimab at three, six, nine and 12 months for the loading phase and interval-matched cohorts. VA; visual acuity. ETDRS; Early Treatment Diabetic Retinopathy Study, VA; visual acuity

The VA followed an uptrending pattern (Fig. 3) from 66.9 ± 13.1 letters at baseline, to 68.5 ± 13.5 letters at 9 months, after which a decrease to 65.9 ± 16.2 was noted at 12 months (Supplemental Digital Content, Table 4). The mean VA increased compared to baseline for both cohorts up to month 9, followed by a decline at 12 months (Fig. 4).

### Changes in macular fluid over time

**Fig. 5:**
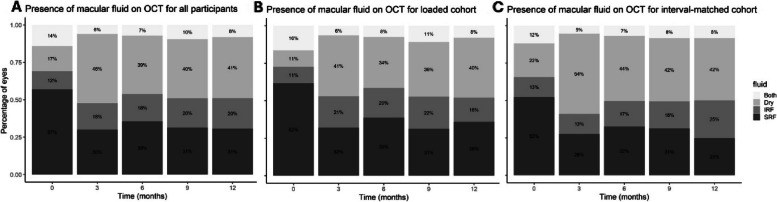
Stacked bar charts illustrating the presence and type of macular fluid at baseline and after switching to faricimab at three, six, nine and twelve months for all eyes treated with faricimab (**5A**), loading cohort (**5B**) and interval-matched cohort (**5C**). IRF; intraretinal fluid, OCT; optical coherence tomography, SRF; subretinal fluid

A significantly greater proportion of eyes displayed a dry macula compared to baseline following faricimab initiation (Fig. 5A): 16.8% at baseline, 46.4% at 3 months, 39.0% at 6 months, 40.0% at 9 months and 40.8% at 12 months (Supplemental Digital Content, Table 4). Both the loaded and interval-matched cohort eyes had a significantly higher proportion of eyes with a dry macula on OCT consistently from baseline to 12 months of treatment (Fig. 5B and C).

## Adverse effects

68 (28.7%) adverse effects were reported (Table 3). Of these, 9 (3.8%) were classified as serious (*n* = 1 atrial fibrillation causing faricimab discontinuation, *n* = 4 stroke, *n* = 1 corneal epithelial defect, *n* = 1 eye pain and periocular swelling causing faricimab discontinuation, *n* = 1 uveitis causing faricimab discontinuation, *n* = 1 migraine causing faricimab discontinuation), with four leading to treatment discontinuation. There were no incidences of endophthalmitis in our cohort.

**Table 3:** Number and type of adverse effects reported by patients while on faricimab treatment. N; number

Adverse event overview	Number (%)
Adverse events (N, %)	68 (28.7%)
Serious adverse events (N,%)	9 (3.8%)
Adverse events leading to treatment discontinuation* (N,%)	4 (1.7%)
**Patients (*n* = 237)**	
Subjective change in Vision (N, %)	10 (4.2%)
Stroke (N, %)^a^	4 (1.7%)
Atrial fibrillation (N, %)^a^	1 (0.4%)
Migraine	2 (0.8%)
Dizziness	2 (0.8%)
Dry mouth	1 (0.4%)
**Eyes (*n* = 297)**	
Floaters (N, %)	11 (3.7%)
Corneal epithelial defect (N, %)^a^	1 (0.3%)
Photophobia (N, %)	1 (0.3%)
Pain (N, %)	6 (2%)
Flashes (N, %)	3 (1%)
Subconjunctival hemorrhage (N, %)	22 (7.4%)
Eye Abrasion (N, **%)**	2 (0.7%)
Uveitis (N, %) ^a^	1(0.3%)

^a^ denotes the adverse events considered as serious

The most frequently reported adverse events were subconjunctival hemorrhage (*n* = 22 eyes, 7.4%), floaters (*n* = 11 eyes, 3.7%) and subjective changes in vision (*n* = 10 patients, 4.2%), which resolved with no intervention.

## Discussion

This study described the efficacy and safety of intravitreal faricimab therapy in previously treated nAMD eyes of patients attending a large tertiary hospital. Faricimab allowed for less frequent injection administration, while maintaining stable vision and producing a beneficial anatomical response. A significantly greater proportion of eyes were dry with faricimab. The eyes that were dry at baseline were switched to faricimab to allow for extension of the administration interval.

While our study demonstrates significant anatomical improvements, these do not always translate directly into proportional VA gains, likely due to factors such as photoreceptor integrity, baseline VA, and disease chronicity [14]. However, the reduced treatment burden and preserved vision represent meaningful outcomes for patients who have already exhibited suboptimal responses to prior therapies. Further research incorporating patient-reported outcome measures is needed to better assess the functional benefits of these treatments in this patient population.

The switch to faricimab allowed for an extension of the treatment interval for 156 (52.5%) eyes. Reducing the frequency of intravitreal injections is advantageous for both patients and healthcare providers. For patients, it can lead to better quality of life. It decreases the logistical challenges associated with frequent clinic visits and anxiety associated with intravitreal injections, potentially improving long-term adherence. From a healthcare system perspective, extended intervals can lead to better resource allocation and can be more cost effective.

While other studies [15] have described accelerated loading and interval-matching strategies, to the best of the authors' knowledge, this is the first report to investigate the impact of the loading schedule on anatomical and functional outcomes in eyes treated before. Overall, the loaded cohort achieved greater CST reduction (- 27.1µm) compared to the interval-matched cohort (-11.6µm). A higher number of eyes became dry in the loading cohort (+ 46 eyes, 31.5% increase) than in the interval-matched cohort (+ 38 eyes, 25.2% increase). Additionally, the loading cohort gained 1.4 letters, whereas the interval-matched cohort lost 0.9 letters. Based on these findings, the loaded cohort exhibited overall superior outcomes.

Comparison of the two cohorts over time showed that at the 12-month follow-up, the CST of the loaded cohort increased, whereas the interval-matched cohort decreased. This analysis is limited by two factors. Firstly, the sample size at the 12-month follow-up was small: 24 eyes in the interval-matched cohort and 25 eyes in the loaded cohort. Secondly, our 12-month data primarily reflect patients with more severe disease. When we began faricimab treatment, we prioritized patients who had been less responsive to other anti-VEGF treatments, i.e. a patient population with more advanced nAMD compared to those treated later. Hence, further studies with longer follow-up periods are needed to provide more reliable 12-month data on the effect of faricimab on CST and to offer additional insights.

Despite the patients in this study being switch patients, we observed a statistically significant improvement and overall clinical stability in VA (66.9 ± 13.1 at baseline to 67.1 ± 15.5, *p* = 0.016). The interval-matched cohort showed a slight decline in VA (65.9 ± 13.8 to 65.0 ± 17.1, *p* = 0.613), but this change was neither statistically nor clinically significant. In contrast, the loaded cohort had a statistically significant improvement (67.9 ± 12.3 to 69.3 ± 13.4, *p* = 0.002); however, given the small magnitude of change (+ 1.4 letters), this is not considered clinically meaningful, as a gain of fewer than 5 letters is unlikely to result in a perceptible functional benefit for patients [16].

The observed discrepancy between the loaded and interval-matched cohorts could be attributed to differences in injection frequency, which may have led to better disease control in the loaded group. However, given the similar overall functional outcomes, clinicians must weigh the benefit of more frequent injections against the burden of additional treatments, particularly since the VA gains, while statistically significant, may not translate into a meaningful visual improvement.

The stable VA observed in our cohort differs from the letter gains reported in TENAYA and LUCERNE trials [10], however they recruited treatment-naïve participants (+ 5.8 letters in TENAYA, + 6.6 letters in LUCERNE) [10]. These trials also identified a CST reduction by -136.8μm (-38%) in TENAYA and -137.1μm (-38.5%) in LUCERNE, consistent with our findings of downtrending CST, despite our patients not being treatment naïve. Similarly, a meta-analysis of four randomised trials (*n* = 1,486 patients), showed that there was no difference in the VA between faricimab and other anti-VEGF treatments, although the faricimab group exhibited lower CST [17]. The maintenance of vision in our nAMD cohort and others [15, 18] highlights the effectiveness of faricimab in real-world settings.

Our outcomes were comparable to those reported by other real-world studies [19]. One study (*n* = 126 participants) reported a reduction in CST (-11.6μm) after three injections with a stable VA [20]. Another multi-centre study (*n* = 337 eyes) demonstrated a + 0.7 letter (*p*-value = 0.196) improvement in VA and a -25.3μm (*p*-value < 0.001) decrease in CST [21]. A separate study (*n* = 190 eyes) reported an improvement in VA from 0.33 ± 0.32 logMAR to 0.27 ± 0.32 logMAR (*p*-value = 0.002). The CST improved from 312.0 ± 87.0μm to 287.0 ± 71.0μm (*p*-value < 0.001) [22]. Contrary to these results, one study that initiated faricimab (*n* = 130 eyes) discontinued it after 6 months in 77 eyes (59.2%) due to insufficient efficacy in 71 eyes [23].

The greatest proportion of eyes that extended their injection interval with faricimab were those previously receiving 4-weekly anti-VEGF injections (79.2%). In contrast, only 23.9% of eyes that had been on 7–8 weekly anti-VEGF injections were able to further extend their injection interval with faricimab. Eyes that were previously on 4-weekly anti-VEGF injections would have had more active disease at baseline, allowing for a greater therapeutic response with faricimab. Additionally, these eyes may have had disease less responsive to anti-VEGF therapy. Faricimab, a bispecific monoclonal antibody targeting both VEGF-A and angiopoietin-2, may provide added benefit in eyes where angiopoietin-2 plays a significant role in disease pathology. For patients already on longer intervals (7–8 weeks) before switching, the disease would have been more stable and responsive to the anti-VEGF therapy, making it harder to further extend the interval.

Six patients experienced systemic adverse events. One patient (0.4%) reported an episode of atrial fibrillation starting a day after faricimab administration, persisting for six days. This patient had a long-standing history of recurrent atrial fibrillation, with both ablation and cardioversion attempts, and had received three faricimab injections. The rate of atrial fibrillation in our study was lower compared to TENAYA (ClinicalTrials.gov identifier, NCT03823287) (1.5%) and LUCERNE (ClinicalTrials.gov identifier, NCT03823300) (0.9%) trials. While pharmacovigilance data have indicated an increased reporting of atrial fibrillation with anti-VEGF therapies (ROR > 1) [24], our patient had a long-standing history of recurrent atrial fibrillation, including prior ablation and cardioversion attempts. Given this pre-existing condition, it is difficult to establish a causal relationship between faricimab and the arrhythmic episode.

Four patients experienced strokes. The first patient, who had no stroke risk factors, was on bilateral faricimab treatment for nine months. They received 12 injections (six per eye), after which they suffered a transient ischemic attack. Prior to the switch, they received 66 aflibercept injections in the right eye and 112 in the left eye. The second patient had type 2 diabetes, hypertension, and stage 3 chronic kidney disease. They received 29 aflibercept and 1 faricimab injection prior to the stroke. The third patient, with a background of severe small vessel ischemia, received 11 aflibercept and 9 faricimab injections before the stroke. The fourth patient, with a complex cardiology history including a transcatheter aortic valve implantation, pacemaker, hypertension, and stage 3 chronic kidney disease, received 10 aflibercept and 2 faricimab injections before the stroke.

Overall, this patient cohort consisted of older individuals (mean age = 80.7 ± 7.0) with multiple comorbidities, reflecting a typical nAMD population outside of clinical trials. Notably, in the TENAYA and LUCERNE trials, the incidence of nonfatal strokes was lower, 1 (0.3%) and 3 (0.9%) events respectively [10], compared to 4 events (1.7%) in our study. We believe this difference to be attributed to the pre-existing health conditions of the participants included in our study, rather than directly caused by faricimab. This assumption is further supported by findings from a large retrospective database study, which did not identify an increased risk of cerebrovascular disease or all-cause hospitalization following the initiation of intravitreal bevacizumab, ranibizumab, or aflibercept [25]. However, further studies powered to investigate these associations specifically for faricimab are needed.

Another patient with a history of migraines experienced prolonged migraines lasting five weeks and switched back to aflibercept, as they suspected drug-related causality. One retrospective study identified headaches/ migraines as a side effect (12%), however this study used a combination cohort of wet AMD and proliferative diabetic retinopathy [26]. One case report described a patient with a history of episodic migraines experiencing a migraine episode four hours after an intravitreal ranibizumab injection [27]. However, this case involved treatment for macular edema secondary to a nonischemic central retinal vein occlusion.

Three patients experienced severe local adverse reactions. One patient (0.3%) with a history of neurotrophic cornea developed a corneal epithelial defect. A previous study reported that 0.5% of participants developed a corneal epithelial defect the day after intravitreal bevacizumab injection [28]. Additionally, the same study demonstrated in animal experiments that topical bevacizumab hindered corneal epithelial healing. These findings suggest the need for increased vigilance when administering anti-VEGF therapy to patients with underlying corneal pathology.

The second patient (0.3%) developed uveitis. A Medicare claims database reported uveitis occurring at a rate of 0.11% per injection with ranibizumab, bevacizumab, or pegaptanib [29]. Additionally, two cases of hypertensive uveitis following intravitreal faricimab have been documented in the literature [30].

The third patient experienced severe pain and periocular swelling following intravitreal injection. While pain associated with intravitreal injections is generally mild, a prospective study reported that among patients who had received five or more intravitreal injections in total, 4.9% experienced severe pain post-injection [31].

Even though a high proportion of our patients experienced minor adverse events, such as subconjunctival hemorrhage (7.4%) and floaters (3.7%), these events were self-limiting and did not impact patient adherence. These adverse events are consistent with those typically observed with anti-VEGF therapies [32]. Given that all our patients were switch patients, their prior experiences with anti-VEGF injections likely helped them manage expectations and minimize the impact of these minor adverse events. Furthermore, the rates of these adverse events were lower than those reported in the TENAYA and LUCERNE trials [10], where conjunctival hemorrhage was observed in 10.8% and 10.6% of participants, respectively, and floaters were reported in 6.9% of participants in TENAYA. Eye pain was also reported less frequently in our study (2%) compared to TENAYA (4.2%). Dizziness was reported by two patients (0.8%) in our cohort, compared to one case (0.3%) in LUCERNE (reported as presyncope) and none in TENAYA.

In our cohort 60 participants (20.2%) received bilateral faricimab treatment. Data on bilateral treatment are limited, and the summary of product characteristics warns that bilateral treatment could pose a higher risk of systemic adverse effects due to increased overall systemic exposure, as well as bilateral reactions [33]. However, we did not observe an increased incidence of such adverse effects, except for the aforementioned patient who experienced a stroke.

Over the course of one year, only 10 eyes (3.4%) required switching back to aflibercept, indicating that faricimab is generally well tolerated, with a favourable safety profile and sustained efficacy, as evidenced by an acceptably low switch rate.

This study has limitations. Firstly, it is a retrospective, single-centre study, limiting the generalizability of our findings. Secondly, our cohort consisted exclusively of individuals of white ethnicity, which restricts the applicability of our findings to more diverse populations. Future multi-ethnic and multi-center studies are needed to validate these results and explore potential variations in treatment response. Additionally, socioeconomic factors and access to care may influence outcomes in broader populations and should be considered in future research. Moreover, a significant proportion of eyes that were receiving previous anti-VEGF treatments at longer intervals were subsequently interval-matched when switched to faricimab, suggesting potential bias from physicians who might have considered patients' previous administration intervals and adjusted the loading phase accordingly. Additionally, the variability in treatment initiation times and follow-up durations within the study period could impact the consistency of the findings. A future prospective cohort study with standardized treatment initiation and follow-up protocols would provide more robust, generalized results. Finally, variations in real-world treatment adherence, such as missed or delayed appointments, were not tracked or accounted for in this study and may have impacted our results.

## Conclusion

In conclusion, we demonstrated that faricimab produced a beneficial anatomical response, significantly reducing CST and macular fluid. VA remained stable, and faricimab allowed for less frequent injections. The loaded cohort exhibited superior outcomes but required more frequent injections. Despite this, both cohorts achieved good anatomical results, and their functional outcomes were similar. Guided by our results, clinicians may choose the loading protocol for patients active at 4 weeks on a previous medication and reserve the interval-matched protocol for patients already on longer intervals. Faricimab demonstrated short-term safety, with serious adverse effects likely influenced by patients' underlying comorbidities. We recommend that clinicians carefully weigh the risks of increased injection frequency against the benefits of enhanced anatomical response and similar functional outcomes when deciding on the loading regimen.

## Supplementary Information

Supplementary Material 1.

## Data Availability

The datasets used and/or analysed during the current study are available from the corresponding author on reasonable request.

## Abbreviations

anti-VEGF: Anti-vascular endothelial growth factor
CST: Central subfield thickness
ETDRS: Early Treatment of Diabetic Retinopathy Study
EMR: Electronic medical records
IRF: Intraretinal fluid
nAMD: Neovascular age-related macular degeneration
OCT: Optical coherence tomography
SD: Standard deviation
SRF: Subretinal fluid
VA: Visual acuity

## References

[1] Wong WL, Su X, Li X, Cheung CMG, Klein R, Cheng C-Y, et al. Global prevalence of age-related macular degeneration and disease burden projection for 2020 and 2040: a systematic review and meta-analysis. Lancet Glob Health. 2014;2:e106–16. 25104651 10.1016/S2214-109X(13)70145-1

[2] Quartilho A, Simkiss P, Zekite A, Xing W, Wormald R, Bunce C. Leading causes of certifiable visual loss in England and Wales during the year ending 31 March 2013. Eye. 2016;30:602–7. 26821759 10.1038/eye.2015.288 PMC5108547

[3] Kovach JL, Schwartz SG, Flynn HW, Scott IU. Anti-VEGF Treatment Strategies for Wet AMD. J Ophthalmol. 2012;2012:1–7. 10.1155/2012/786870 PMC3317200 22523653

[4] Wykoff CC, Clark WL, Nielsen JS, Brill JV, Greene LS, Heggen CL. Optimizing Anti-VEGF Treatment Outcomes for Patients with Neovascular Age-Related Macular Degeneration. J Manag Care Spec Pharm. 2018;24:S3-15. 29383980 10.18553/jmcp.2018.24.2-a.s3 PMC10408401

[5] Heier JS, Brown DM, Chong V, Korobelnik J-F, Kaiser PK, Nguyen QD, et al. Intravitreal Aflibercept (VEGF Trap-Eye) in wet age-related macular degeneration. Ophthalmology. 2012;119:2537–48. 23084240 10.1016/j.ophtha.2012.09.006

[6] Shahzad H, Mahmood S, McGee S, Hubbard J, Haque S, Paudyal V, et al. Non-adherence and non-persistence to intravitreal anti-vascular endothelial growth factor (anti-VEGF) therapy: a systematic review and meta-analysis. Syst Rev. 2023;12:92. 37269003 10.1186/s13643-023-02261-x PMC10237080

[7] Khanna S, Komati R, Eichenbaum DA, Hariprasad I, Ciulla TA, Hariprasad SM. Current and upcoming anti-VEGF therapies and dosing strategies for the treatment of neovascular AMD: a comparative review. BMJ Open Ophthalmol. 2019;4:e000398. 31909196 10.1136/bmjophth-2019-000398 PMC6936465

[8] GOV.UK. MHRA approves faricimab through international work-sharing initiative. 2022.

[9] Panos GD, Lakshmanan A, Dadoukis P, Ripa M, Motta L, Amoaku WM. Faricimab: transforming the future of macular diseases treatment - a comprehensive review of clinical studies. Drug Des Devel Ther. 2023;17:2861–73. 37746113 10.2147/DDDT.S427416 PMC10516184

[10] Heier JS, Khanani AM, Quezada Ruiz C, Basu K, Ferrone PJ, Brittain C, et al. Efficacy, durability, and safety of intravitreal faricimab up to every 16 weeks for neovascular age-related macular degeneration (TENAYA and LUCERNE): two randomised, double-masked, phase 3, non-inferiority trials. The Lancet. 2022;399:729–40. 10.1016/S0140-6736(22)00010-1 35085502

[11] RCOphth. Ophthalmic Service Guidance Intravitreal injection therapy. London; 2018.

[12] RStudio Team (2024). RStudio: Integrated Development Environment for R. RStudio, PBC, Boston, MA URL http://www.rstudio.com/.

[13] Health Research Authority N. What approvals and decisions do I need? https://www.hra.nhs.uk/approvals-amendments/what-approvals-do-i-need/. https://www.hra.nhs.uk/approvals-amendments/what-approvals-do-i-need/. Accessed 1 Jul 2024.

[14] Kim YM, Kim JH, Koh HJ. Improvement of photoreceptor integrity and associated visual outcome in neovascular age-related macular degeneration. Am J Ophthalmol. 2012;154:164-173.e1. 22541932 10.1016/j.ajo.2012.01.030

[15] Ng B, Kolli H, Ajith Kumar N, Azzopardi M, Logeswaran A, Buensalido J, et al. Real-world data on faricimab switching in treatment-refractory neovascular age-related macular degeneration. Life. 2024;14:193. 38398702 10.3390/life14020193 PMC10890640

[16] Beck RW, Maguire MG, Bressler NM, Glassman AR, Lindblad AS, Ferris FL. Visual acuity as an outcome measure in clinical trials of retinal diseases. Ophthalmology. 2007;114:1804–9. 17908590 10.1016/j.ophtha.2007.06.047

[17] Yen W-T, Wu C-S, Yang C-H, Chen Y-H, Lee C-H, Hsu C-R. Efficacy and safety of intravitreal faricimab for neovascular age-related macular degeneration: a systematic review and meta-analysis. Sci Rep. 2024;14:2485. 38291069 10.1038/s41598-024-52942-3 PMC10827713

[18] Pandit SA, Momenaei B, Wakabayashi T, Mansour HA, Vemula S, Durrani AF, et al. Clinical outcomes of faricimab in patients with previously treated neovascular age-related macular degeneration. Ophthalmol Retina. 2024;8:360–6. 37913992 10.1016/j.oret.2023.10.018

[19] Sharma A, Kumar N, Parachuri N, Loewenstein A, Bandello F, Kuppermann BD. Global experience of faricimab in clinical settings - a review. Expert Opin Biol Ther. 2024;24:263–8. 38551188 10.1080/14712598.2024.2336087

[20] Szigiato A, Mohan N, Talcott KE, Mammo DA, Babiuch AS, Kaiser PK, et al. Short-term outcomes of faricimab in patients with neovascular age-related macular degeneration on prior Anti-VEGF therapy. Ophthalmol Retina. 2024;8:10–7. 37673396 10.1016/j.oret.2023.08.018

[21] Khanani AM, Aziz AA, Khan H, Gupta A, Mojumder O, Saulebayeva A, et al. The real-world efficacy and safety of faricimab in neovascular age-related macular degeneration: the TRUCKEE study – 6 month results. Eye. 2023;37:3574–81. 37173428 10.1038/s41433-023-02553-5 PMC10686385

[22] Leung EH, Oh DJ, Alderson SE, Bracy J, McLeod M, Perez LI, et al. Initial real-world experience with faricimab in treatment-resistant neovascular age-related macular degeneration. Clin Ophthalmol. 2023;17:1287–93. 37181079 10.2147/OPTH.S409822 PMC10167970

[23] Kataoka K, Itagaki K, Hashiya N, Wakugawa S, Tanaka K, Nakayama M, et al. Six-month outcomes of switching from aflibercept to faricimab in refractory cases of neovascular age-related macular degeneration. Graefe’s Archive Clin Exper Ophthalmol. 2024;262:43–51. 10.1007/s00417-023-06222-x 37668741

[24] Yang JM, Jung SY, Kim MS, Lee SW, Yon DK, Shin J II, et al. Cardiovascular and cerebrovascular adverse events associated with intravitreal anti-VEGF monoclonal antibodies. Ophthalmology. 2025;132:62–78. 39004231 10.1016/j.ophtha.2024.07.008

[25] Maloney MH, Payne SR, Herrin J, Sangaralingham LR, Shah ND, Barkmeier AJ. Risk of systemic adverse events after intravitreal bevacizumab, ranibizumab, and aflibercept in routine clinical practice. Ophthalmology. 2021;128:417–24. 32781110 10.1016/j.ophtha.2020.07.062

[26] Chiang H, Kim BY. Retrospective analysis of complications from anti-VEGF intravitreal injections using two techniques at a single institution. Invest Ophthalmol Vis Sci. 2015;56:190.

[27] Lerebours VC, Nguyen T-G, Sarup V, Rossi F, Shaikh S. Intravitreal injection-induced migraine headaches. Cureus. 2016;8:e561. 27190726 10.7759/cureus.561 PMC4859816

[28] Colombres GA, Gramajo AL, Arrambide MP, Juarez SM, Arevalo JF, Bar J, et al. Delayed corneal epithelial healing after intravitreal bevacizumab: a clinical and experimental study. J Ophthalmic Vis Res. 2011;6:18–25. 22454702 PMC3306063

[29] Day S, Acquah K, Mruthyunjaya P, Grossman DS, Lee PP, Sloan FA. Ocular complications after anti-vascular endothelial growth factor therapy in medicare patients with age-related macular degeneration. Am J Ophthalmol. 2011;152:266–72. 21664593 10.1016/j.ajo.2011.01.053 PMC3143287

[30] Palmieri F, Younis S, BedanHamoud A, Fabozzi L. Uveitis following intravitreal injections of faricimab: a case report. Ocul Immunol Inflamm. 2024;32:1873–7. 38133943 10.1080/09273948.2023.2293925

[31] Mekala S, Dhoble P, Vishwaraj C, Khodifad AM, Hess OM, Lavanya G. Subjective and objective measures of the patient experience before, during, and after intravitreal anti–vascular endothelial growth factor injections. Indian J Ophthalmol. 2021;69:890–4. 33727454 10.4103/ijo.IJO_1269_20 PMC8012954

[32] Ma P, Pan X, Liu R, Qu Y, Xie L, Xie J, et al. Ocular adverse events associated with anti-VEGF therapy: A pharmacovigilance study of the FDA adverse event reporting system (FAERS). Front Pharmacol. 2022;13:1017889. 36467087 10.3389/fphar.2022.1017889 PMC9716077

[33] Roche. Vabysmo: Summary of product characteristics. 2022.

