# Supplementary material for: Real-world experience of intravitreal faricimab injection in previously treated neovascular age-related macular degeneration eyes: a case series
Source: BMC Ophthalmol. 2025 Mar 10;25:117. doi: 10.1186/s12886-025-03953-9 (PMC11892307; doi:10.1186/s12886-025-03953-9)
Supplement: Supplementary file 1 — Supplementary Material 1. [file 12886_2025_3953_MOESM1_ESM.docx]

**Supplemental Digital Content**

**Figure 1.** Flowchart of the reasons for participant exclusion from the analysis.

**
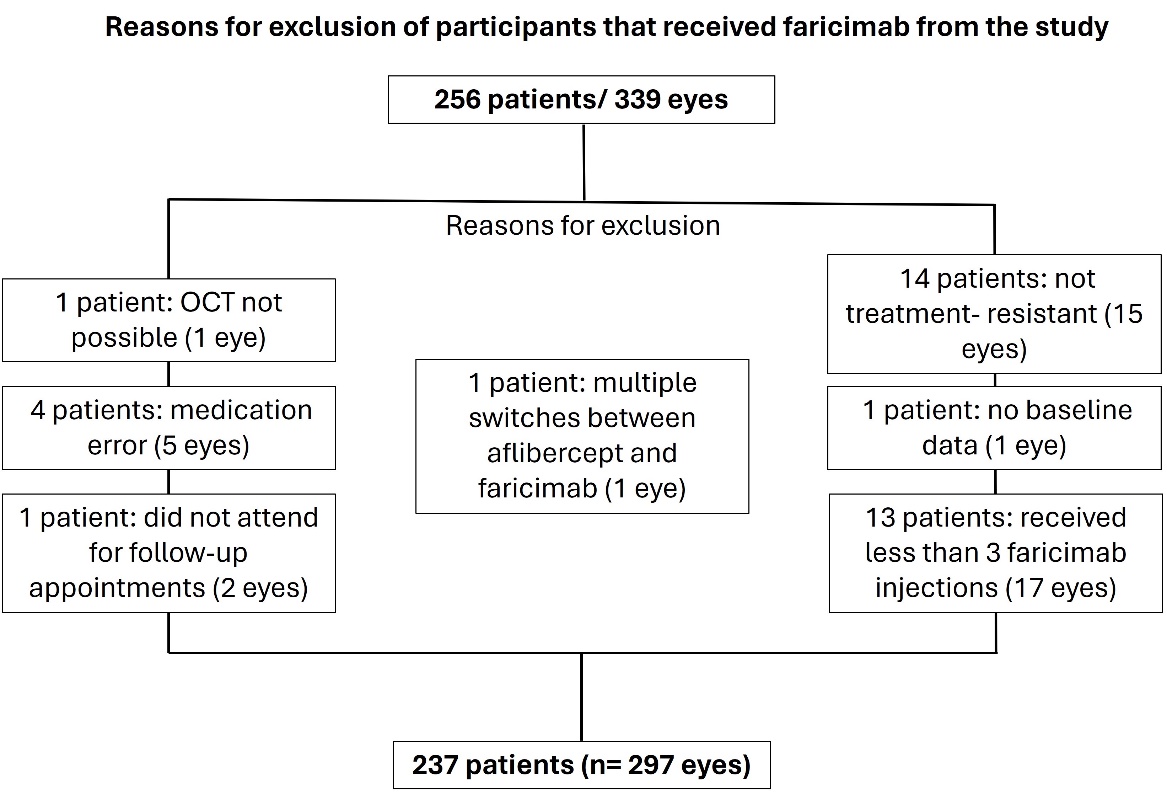
**

**Table 1**. Comparison of injection intervals before and after switch to faricimab, categorised by previous anti-VEGF administration frequency. This analysis utilized the final recorded injection interval for both the previous anti-VEGF treatment and faricimab. Anti-VEGF; anti–vascular endothelial growth factor therapy, N; number.

| Categories according to last anti-VEGF interval | Loading status  (N, %) | Prior anti-VEGF interval (N, %) | Faricimab injection interval (N, %) | P-value |
| --- | --- | --- | --- | --- |
| 4 weekly | Loaded: 45 (58.4%)  Interval-matched: 32 (41.6%) | 77 (25.9%) | 51 (17.1%) | 0.0216 |
| 5 and 6 weekly | Loaded: 77 (54.6%)  Interval-matched: 64 (45.4%) | 141 (47.5%) | 101 (34%) | 0.0101 |
| 7 and 8 weekly | Loaded: 20 (43.5%)  Interval-matched: 26 (56.5%) | 46 (15.5%) | 65 (21.9%) | 0.0700 |
| >8 weekly | Loaded: 4 (12.1%)  Interval-matched: 29 (87.9%) | 33 (11.1%) | 80 (26.9%) | <0.0001 |

**Table 2.** Breakdown of number of eyes administered faricimab at different injection intervals, according to the previous anti-VEGF administration frequency. This analysis utilized the final recorded injection interval for both the previous anti-VEGF treatment and faricimab. Anti-VEGF; anti–vascular endothelial growth factor therapy, N; number.

| Categories according to last faricimab administration frequency | Number of eyes (%) |
| --- | --- |
| Previous anti-VEGF injection interval: 4 weekly (77 eyes) | |
| 4 weekly | 16 (20.8%) |
| 5 to 6 weekly | 27 (35.1%) |
| 7 to 8 weekly | 15 (19.5%) |
| >8 weekly | 19 (24.7%) |
| Previous anti-VEGF injection interval: 5 and 6 weekly (141 eyes) | |
| 4 weekly | 40 (28.4%) |
| 5 to 6 weekly | 54 (38.3%) |
| 7 to 8 weekly | 24 (17%) |
| >8 weekly | 33 (23.4%) |
| Previous anti-VEGF injection interval: 7 and 8 weekly (46 eyes) | |
| 4 weekly | 5 (10.9%) |
| 5 to 6 weekly | 13 (28.3%) |
| 7 to 8 weekly | 17 (37%) |
| >8 weekly | 11 (23.9%) |
| Previous anti-VEGF injection interval: > 8 weekly (33 eyes) | |
| 4 weekly | 0 (nil) |
| 5 to 6 weekly | 7 (21.2%) |
| 7 to 8 weekly | 9 (27.3%) |
| >8 weekly | 17 (51.5%) |

**Table 3**. Comparison of baseline to last documented follow-up anatomical and functional responses to faricimab for all eyes, and according to loading status. CST; central subfield thickness, N; number, VA; visual acuity.

| Parameter | Overall (n=297 eyes) | | |
| --- | --- | --- | --- |
|  | **Baseline** | **Last follow-up** | **p-value** |
| CST (µm) | 325.2± 97.2 | 291.5± 74.4 | <0.0001 |
| VA | 66.9±13.1 | 67.1±15.5 | 0.0158 |
| Dry (N, %) | 50 (16.8%) | 134 (45.1%) | <0.0001 |
| Treatment interval (weeks) | 6±2.3 | 7.2±2.8 | <0.0001 |
|  | **Loaded (n=146 eyes)** | | |
| CST (µm) | 315.1±86 | 288±63.6 | <0.0001 |
| VA | 67.9±12.3 | 69.3±13.4 | 0.00243 |
| Dry (N, %) | 16 (11%) | 62 (42.5%) | <0.0001 |
| Treatment interval (weeks) | 5.3±1.3 | 6.4±2.1 | <0.0001 |
|  | **Interval-matched (n= 151 eyes)** | | |
| CST (µm) | 302.8±57.4 | 291.2±62.6 | 0.0011 |
| VA | 65.9±13.8 | 65±17.1 | 0.613 |
| Dry (N, %) | 34 (22.5%) | 72 (47.7%) | <0.0001 |
| Treatment interval (weeks) | 6.6±2.8 | 7.9±3.2 | <0.0001 |

**Table 4**: Comparison of baseline to follow-up anatomical and functional responses at three, six, nine and twelve months of faricimab treatment for all eyes, loaded cohort and interval-matched cohort eyes. CST; central subfield thickness, N; number, OCT; Optical coherence tomography, VA; visual acuity.

| Overall | | | | | |
| --- | --- | --- | --- | --- | --- |
|  | Baseline | 3 months | 6 months | 9 months | 12 months |
| Number of eyes with OCT available | 297 | 250 | 223 | 157 | 49 |
| CST (µm) | 313.8 ± 80.2 | 292.3±71.1 | 294.8±78.4 | 302.1±68.7 | 295.2±65.2 |
| P-value |  | <0.0001 | 0.0002 | 0.0929 | 0.0765 |
| Dry (N, %) | 50 (16.8%) | 116 (46.4%) | 87 (39%) | 62 (40%) | 20 (40.8%) |
| P-value |  | <0.0001 | <0.0001 | <0.0001 | 0.0002 |
| Number of eyes with VA available | 297 | 270 | 264 | 186 | 64 |
| VA | 66.9±13.1 | 67.7±13.8 | 67.8±14 | 68.5±13.5 | 65.9±16.2 |
| P-value |  | 0.3497 | 0.2790 | 0.1441 | 0.9326 |
| Loading phase cohort | | | | | |
| Number of eyes with OCT available | 146 | 138 | 106 | 74 | 25 |
| CST (µm) | 315.1±86 | 295.7±81.3 | 302.5±92.5 | 305.3±69.4 | 308.8±70.9 |
| P-value |  | 0.001 | 0.0092 | 0.213 | 0.538 |
| Dry (N, %) | 16 (11%) | 56 (40.6%) | 36 (34%) | 27 (36.5%) | 10 (40%) |
| P-value |  | <0.0001 | <0.0001 | <0.0001 | 0.0006 |
| Number of eyes with VA available | 146 | 145 | 130 | 90 | 33 |
| VA | 67.9±12.3 | 68.6±13.2 | 68.4±14.1 | 69.1 ±12.9 | 67.3±13.8 |
| P-value |  | 0.501 | 0.511 | 0.461 | 0.913 |
| Interval-matched cohort | | | | | |
| Number of eyes with OCT available | 151 | 112 | 117 | 83 | 24 |
| CST (µm) | 302.8±57.4 | 288.1±56.3 | 297.8±62.4 | 299.3±68.4 | 281.6±57.3 |
| P-value |  | 0.0186 | 0.0115 | 0.255 | 0.0437 |
| Dry (N, %) | 34 (22.5%) | 60 (53.6%) | 51 (43.6%) | 35 (42.2%) | 10 (41.7%) |
| P-value |  | <0.0001 | 0.0004 | 0.0027 | 0.0792 |
| Number of eyes with VA available | 151 | 125 | 134 | 96 | 31 |
| VA | 65.9±13.8 | 66.7±14.3 | 67.1±13.9 | 68±14.1 | 64.5±18.6 |
| P-value |  | 0.541 | 0.377 | 0.181 | 0.8898 |
